# 慢性淋巴细胞白血病患者COVID-19的单中心报告

**DOI:** 10.3760/cma.j.cn121090-20240621-00230

**Published:** 2024-10

**Authors:** 萧 路, 玲 高, 思褀 钱, 罗梦佳 戴, 子元 周, 彤璐 邱, 奕 夏, 祎 缪, 姝超 秦, 磊 范, 卫 徐, 建勇 李, 华渊 朱

**Affiliations:** 1 南京医科大学第一附属医院，江苏省人民医院血液科，南京 210029 Department of Hematology, The First Affiliated Hospital of Nanjing Medical University, Jiangsu Province Hospital, Nanjing 210029, China; 2 南京医科大学附属宿迁第一人民医院，江苏省人民医院宿迁分院血液科，宿迁 223999 Department of Hematology, The Affiliate Suqian First People's Hospital of Nanjing Medical University, Suqian Branch of Jiangsu Provincial People's Hospital, Suqian 223999, China

**Keywords:** 白血病，淋巴细胞，慢性，B细胞, 新型冠状病毒感染, 新型冠状病毒, 奥密克戎, 布鲁顿酪氨酸酶抑制剂, CD20单抗, Leukemia, lymphocytic, chronic, B-cell, COVID-19, SARS-CoV-2, Omicron, BTK inhibitors, Anti-CD20-based treatments

## Abstract

**目的:**

研究旨在了解中国慢性淋巴细胞白血病（CLL）患者、新型冠状病毒感染（COVID-19）的临床特征、感染风险、转归及疫苗接种情况。

**方法:**

回顾性分析2022年11月至2023年2月在南京医科大学第一附属医院血液科就诊的328例首次确诊COVID-19的CLL患者的临床资料，应用二元Logistic回归模型进行重/危重症COVID-19的单因素、多因素分析。

**结果:**

患者中位年龄为60（24～87）岁，23.5％（77/328）的患者为重/危重症COVID-19。在单因素分析中，年龄>67岁（*OR*＝2.15，95％ *CI* 1.24～3.73，*P*＝0.006）、合并糖尿病（*OR*＝2.09，95％ *CI* 1.05～4.20，*P*＝0.037）、慢性乙型肝炎（*OR*＝2.91，95％ *CI* 1.30～6.51，*P*＝0.010）、CLL进展状态（*OR*＝3.79，95％ *CI* 1.57～9.15，*P*＝0.003）和确诊COVID-19之前3个月内使用CD20单抗治疗（*OR*＝2.79，95％ *CI* 1.35～5.77，*P*＝0.006）是重/危重症COVID-19的危险因素；在多因素分析中，CLL进展状态（*OR*＝2.98，95％ *CI* 1.10～8.10，*P*＝0.033）是重/危重症COVID-19的独立危险因素，另外，接受单药布鲁顿酪氨酸激酶抑制剂（BTKi）可能使重/危重症COVID-19风险减低（*OR*＝0.38，95％ *CI* 0.16～0.90，*P*＝0.028）。对其中242例患者进一步随访至2023年10月，9.1％（22/242）患者在后续多次感染（≥3次），2.1％（5/242）患者持续感染。患者体内新型冠状病毒抗体IgG滴度在感染后3～4个月达峰值（中位值：3.511 S/CO对1.047 S/CO，*P*<0.05）；免疫球蛋白IgA在COVID-19后逐渐降低，且在2～4周至低谷（中位值：0.30 g/L对0.74 g/L，*P*<0.05）。整体队列中COVID-19合并CLL病死率为2.7％（9/328），主要死亡原因为呼吸衰竭和心源性猝死。

**结论:**

CLL患者新型冠状病毒疫苗接种率较低，重/危重症COVID-19比例高。CLL疾病进展是CLL患者发生重/危重症COVID-19的独立危险因素；3个月内CD20单抗治疗是加重COVID-19的危险因素，而持续的BTKi治疗可能减轻重/危重症COVID-19的风险。

## Abstract (en)

**Objective:**

To investigate the vaccination status, characteristics and prognosis of patients suffering from a combination of COVID-19 and chronic lymphocytic anemia（CLL）in China.

**Methods:**

Clinical data of 328 patients with chronic lymphocytic leukemia（CLL）who were first diagnosed with COVID-19 and treated in the Department of Hematology of Jiangsu Provincial People's Hospital between November 2022 and February 2023 were retrospectively analyzed. Univariate and multivariate analysis of data of patients with severe/critical COVID-19 were conducted by applying the binary logistic regression model.

**Results:**

The median age of the CLL patients was 60（24–87）years. 23.5%（77/328）of these patients suffered from severe/critical COVID-19 infection. Univariate analysis of the data demonstrated that a combination of factors including age >67 years（*OR*＝2.15, 95% *CI* 1.24–3.73, *P*＝0.006）, diabetes（*OR*＝2.09, 95% *CI* 1.05–4.20, *P*＝0.037）, chronic hepatitis B（*OR*＝2.91, 95% *CI* 1.30–6.51, *P*＝0.010）, CLL progressive（*OR*＝3.79, 95% *CI* 1.57–9.15, *P*＝0.003）and CD20 antibody-based treatments within three months prior to the COVID-19 infection（*OR*＝2.79, 95% *CI* 1.35–5.77, *P*＝0.006）were the risk factors for severe/critical COVID-19. According to the multivariate analysis, CLL progressive（*OR*＝2.98, 95% *CI* 1.10–8.10, *P*＝0.033）was an independent risk factor for severe/critical COVID-19 and administration of the BTK（Bruton tyrosine kinase）inhibitor monotherapy might exert a protective effect and influence a positive outcome of the COVID-19 infection（*OR*＝0.38, 95% *CI* 0.16–0.90, *P*＝0.028）. Among the 242 patients who were followed up until October 2023, 9.1%（22/242）had multiple subsequent COVID-19 infections（≥3）, and 2.1%（5/242）had persistent COVID-19 infections（patients with persistent positive test for the SARS-CoV-2 antigen testing until missing follow-up for any reason）. The peak value of the anti-SARS-CoV-2-IgG titres was observed between three and four months post symptom onset（median: 3.511 S/CO *vs* 1.047 S/CO, *P*<0.05）. The levels of immunoglobulin A gradually decreased following infection with COVID-19, and its trough levels were attained between two to four weeks post infection（median: 0.30 g/L *vs* 0.74 g/L, *P*<0.05）. According to this study the mortality of patients suffering from a combination of COVID-19 infection and CLL was 2.7%（9/328）, and the main reason for their death was respiratory failure and heart failure.

**Conclusion:**

A low rate of COVID-19 vaccination and a high rate of severe/critical COVID-19 infection was observed in the CLL patients. CLL progressive was associated with severe/critical COVID-19. Anti-CD20-based treatments received within the past three months might be a risk factor for exacerbation of COVID-19 infection, whereas a monotherapy with BTK inhibitors exert a protective effect and improve outcome of COVID-19 infection.

慢性淋巴细胞白血病/小淋巴细胞淋巴瘤（CLL/SLL）是一种共表达CD5和CD23的单克隆成熟小B淋巴细胞增殖性疾病，以单克隆淋巴细胞在外周血、骨髓、淋巴结和脾脏聚集为特征，是一组与免疫缺陷显著相关的恶性肿瘤[1]。随着新型冠状病毒（SARS-CoV-2）（以下简称新冠病毒）的Omicron变异株在世界范围内的传播，CLL患者由于自身免疫缺陷（如细胞免疫功能障碍和补体激活受损）而处于高风险感染中，且各种治疗进一步抑制了自身免疫功能，导致其新冠病毒疫苗接种的保护率低于正常人群[2]–[3]。既往回顾性研究显示CLL患者的新型冠状病毒感染（COVID-19）临床症状重且死亡率高[4]–[6]。然而，我国关于CLL患者COVID-19的临床特征、疫苗接种、重/危重症COVID-19的风险因素、结局转归、再感染以及感染后实验室指标变化的数据有限。为了研究和优化该类患者的长期管理，我们回顾性分析2022年11月至2023年2月在南京医科大学第一附属医院血液科就诊的CLL患者，并进一步随访确诊COVID-19的CLL患者至2023年10月，描述了Omicron病毒株流行期间感染的临床特征和结局。

## 病例与方法

1. 病例：本研究共纳入328例2022年11月至2023年2月在南京医科大学第一附属医院血液科就诊的首次发生COVID-19的CLL患者，所有纳入CLL患者初诊时均行外周血流式细胞术免疫表型检测，诊断标准参照国际慢性淋巴细胞白血病工作组2018指南[7]。所有患者均通过新冠病毒抗原检测法或新冠病毒核酸检测法为阳性，其中270例患者于医院就诊通过新冠病毒抗原检测或新冠病毒核酸检测法为阳性，58例患者居家自行新冠病毒抗原检测为阳性。本研究经南京医科大学第一附属医院伦理委员会批准（批件号：2024-SR-619）。

2. 临床资料：采取回顾性统计、问卷及电话随访调研，调研内容包括：患者首次感染前特征（年龄、性别、合并疾病包括高血压、糖尿病、慢性乙型肝炎、慢性心脏疾病、慢性肾脏疾病、慢性肺部疾病、肥胖及其他肿瘤病史、新冠病毒疫苗接种、CLL状态、抗肿瘤治疗）、首次COVID-19临床症状、临床严重程度、抗COVID-19治疗、护理环境和转归、感染情况、感染后的实验室指标变化等。合并疾病信息均来自患者既往病史记录或患者所填问卷调查。

3. 症状评估：根据《新型冠状病毒感染诊疗方案（试行第十版）》[8]对入院时COVID-19严重程度进行分级：轻型或中型（非肺炎和轻度肺炎）、重型［呼吸困难、呼吸频率≥30次/min、血氧饱和度（SPO_2_）≤93％、氧合指数（PaO_2_/FiO_2_）≤300 mmHg（1 mmHg＝0.133 kPa）或肺部影像学显示24～48 h内病灶明显进展>50％］和危重型（呼吸衰竭、感染性休克或多器官功能障碍或衰竭）。

4. 随访：得到患者知情同意后，通过住院病历及门诊病历系统获取患者基本情况及实验室结果，并通过问卷及电话随访进一步调研，随访截止时间为2023年10月30日，中位随访时间10.3（1.7～12.1）个月。

5. 统计学处理：统计分析采用SPSS 26.0软件，计数资料以例数和百分比表示，并使用*χ*^2^检验进行差异性分析。计量资料以*M*（范围）表示。应用二元Logistic回归模型进行重/危重症COVID-19预测危险因素的单因素、多因素分析。通过受试者工作特性曲线分析得出与重/危重症COVID-19最相关的年龄的Cut-off值为67岁，故以67岁为界值进行年龄分组。*P*<0.05为差异有统计学意义。

## 结果

1. CLL患者确诊COVID-19前的临床特征：2022年11月至2023年2月期间328例CLL患者首次感染COVID-19。中位年龄为60（24～87）岁，整个队列的基线特征如表1所示。感染COVID-19前接种过新冠病毒疫苗的患者比例为39.7％（129/325），其中91.5％（118/129）患者接种灭活疫苗（Vero cell），7.0％（9/129）接种重组亚单位蛋白疫苗（CHO cell）和1.5％（2/129）接种腺病毒载体疫苗。本队列中，在确诊COVID-19时36.6％（120/328）患者处于观察等待期（未达CLL治疗指征），63.4％（208/328）患者既往接受了针对CLL的治疗，其中42.4％（139/328）患者处于一线治疗、12.8％（42/328）处于二线治疗和8.2％（27/328）患者处于多线（≥三线）治疗；197例完成了CLL疗效评估；183例在确诊COVID-19前3个月内正接受针对CLL治疗：108例接受布鲁顿酪氨酸激酶抑制剂（BTKi）单药治疗，其中伊布替尼51例、泽布替尼28例、奥布替尼21例、阿卡替尼7例、LOXO-305 1例；18例患者接受BCL2抑制剂（BCL2i）单药治疗；15例接受OFCG（奥布替尼+氟达拉滨+环磷酰胺+奥妥珠单抗）方案；12例接受BTKi联合CD20单抗治疗；8例接受BTKi联合BCL2i治疗，22例接受以CD20单抗为基础的化疗。

**表1:** COVID-19的328例慢性淋巴细胞白血病患者的临床特征

临床特征	例（%）
性别	
女	117（35.7）
男	211（64.3）
年龄	
≤67岁	244（74.4）
>67岁	84（25.6）
感染COVID-19前合并疾病	
无	163（49.7）
1种合并疾病	100（30.5）
2种合并疾病	45（13.7）
3种及以上合并疾病	20（6.1）
高血压	81（24.7）
糖尿病	41（12.5）
慢性乙型肝炎	27（8.2）
慢性心脏病	19（5.8）
慢性肾脏疾病	10（3.0）
慢性肺部疾病	4（1.2）
肥胖（BMI>30）	8（2.4）
吸烟史	48（14.6）
其他肿瘤病史	13（4.0）
CLL疾病状态（COVID-19诊断时）	
观察等待期	120（36.6）
CR	90（27.4）
PR	46（14.0）
SD	39（11.9）
PD	22（6.7）
未疗效评估	11（3.4）
COVID-19前的CLL治疗线数	
观察等待期	120（36.6）
一线	139（42.4）
二线	42（12.8）
三线及以上	27（8.2）
接种新冠病毒疫苗针剂	
未接种过	196（60.3）
1针剂	10（3.1）
2针剂	44（13.5）
3针剂	73（22.5）
4针剂	2（0.6）
接种新冠病毒疫苗剂型	
灭活疫苗	118（91.5）
北京科兴中维	61（47.3）
国药北京生物	38（29.4）
武汉生物	2（1.6）
兰州生物	2（1.6）
长春生物	10（7.7）
成都生物	2（1.6）
深圳康泰	3（2.3）
重组亚单位蛋白疫苗	9（7.0）
安徽智飞龙科马	9（7.0）
腺病毒载体疫苗	2（1.5）
康希诺	2（1.5）
COVID-19严重程度	
轻症	251（76.5）
重症	68（20.7）
危重症	9（2.8）
COVID-19症状	
发热	251（76.5）
肺部症状	221（67.4）
肺外症状	85（25.9）
无症状	22（6.7）
COVID-19管理	
门诊治疗	245（74.7）
住院治疗	83（25.3）
重症监护病房	9（2.7）
流量氧疗	47（14.3）
有创机械通气	3（0.9）

**注** COVID-19：新型冠状病毒感染；BMI：身体质量指数；CR：完全缓解；PR：部分缓解；SD：疾病稳定；PD：疾病进展

2. CLL患者首次确诊COVID-19后的临床特征：本队列中轻/中症COVID-19、重症COVID-19和危重症COVID-19患者比例分别为76.5％（251/328）、20.7％（68/328）和2.8％（9/328）。43例轻/中症COVID-19患者因口服抗新冠病毒药物而暂停CLL治疗方案：35例暂停BTKi、8例暂停BCL2i；中位停药时间为10（2～50）d，后均恢复用药。30例重/危重症COVID-19患者因感染症状重，在积极治疗COVID-19期间予暂停当前CLL治疗方案：23例暂停BTKi、3例暂停BCL2i、2例暂缓BTKi联合BCL2i及CD20单抗和2例暂缓OFCG方案治疗；中位停药时间为30（5～120）d，1例患者SARS-CoV-2持续感染（至CLL进展前新冠抗原未转阴），停用泽布替尼+维奈克拉20 d后发生CLL进展，排除5例死亡患者，均恢复用药；6例重症COVID-19患者予BTKi减量处理：其中4例伊布替尼由原剂量420 mg/d改为280 mg/d，2例奥布替尼由原剂量150 mg/d改为50 mg/d，中位减量天数为16.5（3～55）d，后均恢复原剂量。

3. 重/危重症COVID-19相关因素分析：在单因素分析中，年龄>67岁（*OR*＝2.15，*P*＝0.006）、合并糖尿病（*OR*＝2.09，*P*＝0.037）、合并慢性乙型肝炎（*OR*＝2.91，*P*＝0.010）、CLL疾病进展（PD）（*OR*＝3.79，*P*＝0.003）、确诊COVID-19之前3个月内使用CD20单抗治疗（*OR*＝2.79，*P*＝0.006）与重/危重症COVID-19有关。在多因素分析中，CLL PD（*OR*＝2.98，*P*＝0.033）是重/危重症COVID-19的独立危险因素，另外，接受BTKi单药治疗（*OR*＝0.38，*P*＝0.028）可能使重/危重症COVID-19风险减低（表2）。

**表2:** 重/危重症COVID-19的相关因素的单因素、多因素分析

因素	单因素分析		多因素分析	
	*OR*（95% *CI*）	*P*值	*OR*（95% *CI*）	*P值*
性别	1.04（0.61~1.77）	0.899		
女				
男				
年龄	2.15（1.24~3.73）	0.006	1.64（0.72~3.78）	0.242
≤67岁				
>67岁				
COVID-19前合并疾病				
无	0.80（0.48~1.34）	0.395		
1种合并疾病	0.75（0.42~1.33）	0.326		
2种合并疾病	1.58（0.79~3.15）	0.196		
3种以上合并疾病	2.31（0.91~5.88）	0.079		
高血压	1.42（0.80~2.51）	0.230		
糖尿病	2.09（1.05~4.20）	0.037	1.61（0.61~4.30）	0.339
慢性乙型肝炎	2.91（1.30~6.51）	0.010	1.62（0.50~5.24）	0.423
慢性心脏病	1.55（0.57~4.22）	0.394		
慢性肾脏疾病	2.24（0.62~8.14）	0.222		
慢性肺部疾病	1.09（0.11~10.61）	0.942		
肥胖（BMI>30）	0.50（0.06~4.19）	0.526		
吸烟史	1.25（0.63~2.51）	0.524		
其他肿瘤病史	0.58（0.13~2.68）	0.487		
接种新冠病毒疫苗针剂				
未接种过	1.39（0.82~2.38）	0.225		
1针剂	1.40（0.35~5.53）	0.635		
2针剂	0.81（0.37~1.76）	0.588		
≥3针剂	0.68（0.36~1.30）	0.245		
CLL疾病状态（COVID-19诊断时）				
观察等待期	0.59（0.33~1.03）	0.065		
CR	0.95（0.53~1.70）	0.850		
PR	0.80（0.36~1.74）	0.566		
SD	1.38（0.65~2.93）	0.404		
PD	3.79（1.57~9.15）	0.003	2.98（1.10~8.10）	0.033
COVID-19前的CLL治疗线数				
观察等待期	0.55（0.32~0.96）	0.036		
一线	1.18（0.71~1.97）	0.523		
二线	1.56（0.76~3.17）	0.224		
三线及以上	1.71（0.74~3.99）	0.211		
CLL治疗（COVID-19确诊后3个月内）				
BTKi单药	0.37（0.19~0.71）	0.003	0.38（0.16~0.90）	0.028
BTKi+anti-CD20	1.17（0.48~2.86）	0.738		
BTKi+BCL2i	2.85（0.69~11.86）	0.150		
BTKi+anti-CD20+BCL2i	8.56（0.87~84.29）	0.066		
BCL2i	0.51（0.14~1.85）	0.307		
anti-CD20	3.83（0.83~17.75）	0.086		
anti-CD20+BCL2i	2.78（0.38~20.24）	0.314		
苯达莫司汀+anti-CD20	2.09（0.45~9.70）	0.345		
CLL既往治疗（COVID-19确诊前）				
anti-CD20	2.79（1.35~5.77）	0.006	1.69（0.63~4.48）	0.296
BCL2i	1.72（0.77~3.86）	0.186		

**注** COVID-19：新型冠状病毒感染；BMI：身体质量指数；CR：完全缓解；PR：部分缓解；SD：疾病稳定；PD：疾病进展；BTKi：布鲁顿酪氨酸激酶抑制剂；anti-CD20：CD20单抗；BCL2i：BCL2抑制剂

4. 感染前后实验室指标变化：收集到61例COVID-19患者在感染前、感染后2周内、感染后2～4周、感染后4～8周外周血血象（ANC、绝对淋巴细胞计数、HGB、PLT）：41例轻症及20例重/危重症COVID-19患者队列中的外周血象变化差异无统计学意义（*P*>0.05）；PD队列（6例）中，ANC在感染后2～4周升高（中位值：6.34×10^9^/L对2.63×10^9^/L，*P*<0.05），在4～8周后逐渐恢复至感染前（中位值：2.70×10^9^/L对6.34×10^9^/L，*P*<0.05）。另外，15例患者PLT在感染后2周内降低（中位值：53×10^9^/L对97×10^9^/L，*P*<0.001），其中13例在感染后4～8周升高至感染前水平（中位值：90×10^9^/L对53×10^9^/L，*P*<0.001），而2例患者在后续随访中持续降低。新冠病毒疫苗接种、COVID-19多次、随访期间接受化疗、CLL PD、重/危重症COVID-19均与感染后2周内PLT降低无相关性（*P*>0.05）。

22例患者感染前后的新冠病毒抗体滴度变化（感染后1个月内、感染后1～2个月、感染后2～3个月、感染后3～4个月、感染4个月后）：其中新冠病毒抗体IgG（SARS-CoV-2-IgG）滴度在感染后3～4个月达峰值（中位值：3.511 S/CO对1.047 S/CO，*P*＝0.041），在4个月后开始降低（中位值：0.238 S/CO对3.511 S/CO，*P*＝0.040）而SARS-CoV-2-IgM滴度差异无统计学意义（*P*>0.05）。

29例患者感染前后的免疫球蛋白水平变化（感染前、感染后2周内、感染后2～4周、感染后4～8周，感染8周后）：其中免疫球蛋白IgA在感染后降低，于感染后2～4周至低谷，感染后4～8周恢复至感染前（中位值：0.74 g/L对0.30 g/L对0.76 g/L，*P*<0.05），而免疫球蛋白IgG、IgM水平变化差异无统计学意义（*P*>0.05）。

5. 患者COVID-19再次感染情况：对本队列242例患者进一步随访至2023年10月，33.9％（82/242）患者感染2次，5.8％（14/242）感染3次，3.3％（8/242）患者感染多次（>3次），另有2.1％（5/242）患者持续感染（首次新冠病毒抗原检测持续阳性至任何原因失访）。感染次数越多的患者中正在接受周期化疗（基于CD20单抗的免疫化疗）的患者比例也越高［感染1次：6.8％（9/133）；2次：15.9％（13/82）；3次：35.7％（5/14）；>3次：87.5％（7/8）］。

6. COVID-19结局转归：随访至2023年10月，队列COVID-19合并CLL死亡率为2.7％（9/328）（表3），6例危重症，3例重症，其中4例CLL在感染期间病情恶化。44.4％（4/9）死于呼吸衰竭，33.3％（3/9）死于心源性猝死，22.2％（2/9）死于多器官衰竭。

**表3:** 死亡患者队列的临床特征（9例）

临床特征	例数（%）
性别	
女	4（44.4）
男	5（55.6）
年龄	
≤67岁	5（55.6）
>67岁	4（44.4）
COVID-19前合并疾病种数	
0种	3（33.3）
1种	3（33.3）
2种	0（0）
≥3种	3（33.3）
CLL疾病状态（COVID-19诊断时，8例）	
CR	1（11.1）
PR	1（11.1）
SD	2（22.2）
PD	4（44.4）
未疗效评估	1（11.1）
COVID-19前的CLL治疗线数	
观察等待期	0（0）
一线	3（33.3）
二线	1（11.1）
三线及以上	5（55.6）
COVID-19前的CLL治疗（3个月内）	
BTKi单药	1（11.1）
BTKi+anti-CD20	2（22.2）
BTKi+BCL2i	1（11.1）
BTKi+anti-CD20+BCL2i	1（11.1）
BCL2i	1（11.1）
Anti-CD20	2（22.2）
接种新冠病毒疫苗针剂	
未接种过	
1针剂	0（0）
2针剂	1（11.1）
3针剂	2（22.2）
死亡原因	
呼吸衰竭	4（44.4）
心源性猝死	3（33.3）
多器官衰竭	2（22.2）
COVID-19临床严重程度	
重症	3（33.3）
危重症	6（66.6）

**注** COVID-19：新型冠状病毒感染；CR：完全缓解；PR：部分缓解；SD：疾病稳定；PD：疾病进展；BTKi：布鲁顿酪氨酸激酶抑制剂；anti-CD20：CD20单抗；BCL2i：BCL2抑制剂

## 讨论

2020年以来，COVID-19在全球范围内大规模流行[8]，主要流行毒株包括Delta、Omicron等变异株，Omicron变异株致病力较弱，但免疫逃逸和传播力更强，在Omicron变异株流行期及新冠病毒疫苗接种普及后COVID-19病死率显著下降，新冠病毒感染逐渐常态化，但CLL患者由于自身免疫缺陷及长期免疫抑制治疗，CLL患者COVID-19的重症发生率及死亡率仍不可忽视。目前国内COVID-19在CLL患者中的流行病学、临床特点、治疗反应及转归等数据有限，尤其是再感染情况以及感染后实验室指标变化数据尚缺乏。

据EPICOVIDEHA研究[9]报告显示，Omicron变异株感染的血液恶性肿瘤（HMs）患者的总住院率为52.1％，死亡率为8.6％，与非住院患者相比，住院患者中老年人、合并其他疾病、活动性疾病和接受CD20单抗治疗更常见。国外Omicron变异株感染的CLL患者住院率26％，30 d内死亡率为2％[10]。

本队列中，重/危重症COVID-19的发生率为23.5％，总死亡率为2.7％，与重/危重症COVID-19相关的危险因素包括高龄、合并糖尿病、慢性乙型肝炎、CLL进展、CD20单抗治疗，与国外数据相似。9.1％患者多次（≥3次）COVID-19，这也体现了Omicron变异株的免疫逃逸和传播力更强，尤其在血液病患者中。另外，PD患者在COVID-19后ANC升高，提示CLL PD患者在COVID-19后可能容易并发其他细菌感染。另外，本队列中免疫球蛋白IgA含量在COVID-19后出现降低，据Andersen等[11]和Crassini等[12]研究证明，与低水平的IgG或IgM相比，低水平的IgA是更好的严重感染预测因素，因此感染期间需进一步加强免疫及营养。综合以上研究，CLL患者中观察到较高的重症感染率与反复感染率，这凸显了在Omicron变异株流行期间对这些患者实施预防措施的必要性。

多项研究探讨了血液病患者新冠病毒疫苗接种的有效性和安全性[13]–[14]，据报道，HMs患者对新冠病毒疫苗应答水平低、持续时间短、血清转化率低，尤其是CLL和其他惰性B细胞非霍奇金淋巴瘤患者[15]。此外，随着疫苗接种完成后时间的推移，疫苗诱导的免疫保护效果不断下降，患者个体感染的可能性逐渐增加[16]。Vero cell、CHO cell和腺病毒载体疫苗是我国主要许可的疫苗。本队列中，疫苗接种情况在队列分析中与危/重症COVID-19无显著相关性，原因可能是CLL患者对疫苗接种的有效性较低[13]。据国内外数据表明，加强疫苗免疫可能对保护HMs患者免受重症COVID-19尤为重要[17]–[18]。《成人血液病患者新型冠状病毒疫苗接种中国专家共识（2023年版）》[19]建议CLL启动治疗前首选灭活疫苗完善新冠病毒疫苗基础免疫，并尽早完成加强免疫接种。

国外研究报道SARS-CoV-2-IgM滴度在感染后2至5周达到峰值[20]–[21]，3至5周内下降[21]。SARS-CoV-2-IgG滴度在感染后3至7周达到峰值[20]，第8周下降[22]。而国内一项2022年12月至2023年5月Omicron流行期间健康人群的队列研究[23]提示SARS-CoV-2-IgG在感染后1个月滴度达峰值后开始下降，2～3个月进入短暂的平台期，4个月后开始再次急剧下降。另一项健康人群的队列研究[24]中报道SARS-CoV-2-IgG中位滴度峰值为192.21（1.77～247.84）S/CO。本队列中，SARS-CoV-2-IgG滴度在COVID-19后3～4个月达峰值，4个月后开始下降，中位滴度峰值3.51（0.04～214）S/CO。以上差异考虑一方面可能由于纳入研究对象不同，HMs患者由于自身免疫缺陷及长期免疫抑制治疗，导致感染后的SARS-CoV-2抗体滴度低且变化时间趋势不同，另一方面我们采集到的SARS-CoV-2抗体患者例数较少，不能完全代表CLL患者感染后SARS-CoV-2抗体滴度变化。

CLL治疗方面，BTKi是否对CLL患者合并COVID-19有保护作用仍有争议，一方面，BTKi具有继发感染的风险并导致体液免疫受损[25]–[26]，另一方面，BTKi可能减轻对COVID-19的高炎症反应导致的肺损伤[26]–[27]。回顾性研究[28]显示与其他治疗方案相比，伊布替尼并未加重CLL患者COVID-19的症状，且死亡率差异无统计学意义。在本队列中，单药BTKi治疗可能使重症COVID-19风险减低，感染期间予BTKi短暂停药或减量的患者绝大部分均恢复BTKi原剂量用药。综上，COVID-19时，建议结合临床症状以及CLL状态，合并轻/中症COVID-19的患者可继续口服BTKi；重/危重症患者，建议短期停药，待不良反应恢复至≤1级后继续服药。不过应注意药物相互作用，如抗病毒药物利托那韦是一种强CYP3A抑制剂，服用利托那韦期间最好暂时停用BTKi[29]。

CD20单抗可以杀死肿瘤细胞[30]，但也会清除体内的正常B淋巴细胞，导致持久免疫抑制[31]。据报道[9]，CD20单抗治疗会增加重症COVID-19的风险。本队列研究中，过去3个月内基于CD20单抗治疗与重/危重症COVID-19相关。因此，建议接受基于CD20单抗的免疫化疗并在治疗期间COVID-19的CLL患者监测免疫功能并暂停CD20单抗治疗。此外，由于接受其他治疗的患者数量有限，我们无法就它们与重/危重症COVID-19的相关性得出任何结论。

综上所述，本研究总结了在本中心CLL患者SARS-CoV-2 Omicron变异株感染的特征和结果，描述了较高的重症感染率，年龄、合并糖尿病、慢性乙型肝炎、CLL PD是重/危重症感染的危险因素。CD20单抗暴露（3个月内）与较高的重/危重症感染率相关，而单药BTKi可能减轻或不加重COVID-19。Omicron变异株传播力更强，CLL患者易重复多次感染，本研究可为CLL患者在Omicron变异株流行期间的管理预防提供证据和建议。
